# Variation between pragmatic and standardised blood pressure measurements in a Nigerian primary care clinic

**DOI:** 10.4102/safp.v62i1.5035

**Published:** 2020-03-12

**Authors:** Oluwaseun S. Ojo, Ademola O. Egunjobi, Akinfemi J. Fatusin, Bolatito B. Fatusin, Odunola O. Ojo, Babajide A. Taiwo, Ibrahim B. Ghazali, Nurudeen A. Gbadamosi

**Affiliations:** 1 Department of Family Medicine, Federal Medical Center, Abeokuta, Ogun State, Nigeria; 2 Department of Family Medicine, Federal Medical Center, Gusau, Zamfara State, Nigeria; 3 Department of Nursing Sciences, Ogun State School of Nursing, Abeokuta, Ogun State, Nigeria

**Keywords:** usual-care, guideline concordant, blood pressure measurement, family practice clinic, primary care

## Abstract

**Background:**

A significant difference in the blood pressure (BP) value of a patient taken by different health workers has been a subject of discussion among health workers. This study investigated the variations between usual-care and guideline-concordant BP measurement protocols and evaluated the implications of the disparities on diagnosis and treatment decision.

**Methods:**

A cross-sectional study was conducted among 206 participants. The usual-care and guideline-concordant BP readings taken from each participant by the regular clinic nurses and research-trained nurses, respectively, were obtained.

**Results:**

Majority of the regular clinic nurses following the usual-care protocol used the left arm for BP measurement (59.7%). The systolic BP (SBP) and diastolic BP (DBP) readings were higher on the right arm in 55.3% and 39.2% of the participants, respectively. The mean guideline-concordant BP was 7.67 mmHg higher than the mean usual-care for SBP (*p* ≤ 0.05) and 7.14 mmHg higher for DBP (*p* ≤ 0.05). The proportion of participants classified as having hypertension and uncontrolled BP was 11.8% and 15.0% lower when using usual-care BP compared to guideline-concordant BP, respectively. Fifty-one (24.8%) respondents were advised incorrect treatment based on usual-care BP measurement. The Bland-Altman plot showed that limits of agreement were wider than within the 10 mmHg clinical reference range and unacceptable for clinical purposes.

**Conclusion:**

The usual-care and guideline-concordant BP measurement protocols were significantly different, and the disparity had significant consequences on the diagnosis and treatment of hypertension. Health workers should strictly adhere to the guidelines on BP measurement to avoid mismanagement of patients.

## Introduction

Hypertension is an enormous independent risk factor for cardiovascular disease, stroke and renal disease,^1^ and the accurate assessment of blood pressure (BP) is the foundation of hypertension management. The various hypertension treatment guidelines helped derive the target BPs for individuals with different levels of cardiovascular risks from various trials that used a specific standardised method of BP measurement recommended by various hypertension guidelines.^2,3,4^ Thus, it is imperative to comply with this standard measurement protocol in the clinic so as to avert treatment of hypertension at levels different from those recommended by guidelines.

In a typical Nigerian primary care clinic, BP is assessed by health workers as part of an initial or ongoing assessment of patients. Unfortunately, the measurement of BP done by the primary health care workers seldom adheres to the guideline-concordant BP measurement protocol.^5,6,7,8,9^ Hence, the measured BP could substantially vary from the true value. This may result in improper labelling of patients, under-treatment or over-treatment of hypertensive patients, putting them at risk for cardiovascular consequences or exposing them to risk of needless adverse interventions. The BP measurement errors and their consequences may be worse in Nigeria with a low patient-to-health-worker ratio.^10^ In addition, although from anecdotal reports, a significant difference in the BP value of a patient taken by different health workers during routine clinic has also been a subject of discussion among health workers.

Previous Nigerian studies have focused on sphygmo-manometer-related errors in BP measurement,^11,12^ whereas there is a lack of research focusing on the errors in BP measurement because of the protocol used. There is a need to assess the reliability of the protocol used in measuring BP in a busy Nigerian primary care clinic. Considering the lack of comparative data on differences between usual-care (pragmatic) and guideline-concordant (standardised) BP measurement protocols in Nigeria, this study assessed the variations between usual-care and guideline-concordant BP measurement and evaluated the implications of the disparities on diagnosis and treatment decision. The results of this study may sensitise health workers on the need to follow recommended guidelines when measuring BP. This may improve the diagnosis of hypertension and overall treatment outcomes in patients with hypertension.

## Operational definitions

### Usual-care blood pressure measurement

Participants’ BP is measured by a medical or paramedical staff during the usual clinical examination.

### Guideline-concordant blood pressure measurement

Participants’ BP is measured by a trained research staff (nurse) according to the recommended guidelines for taking BP.

## Methods

### Study setting

The study was carried out at the GOPC of a tertiary hospital in south west Nigeria. Nigeria is a country with a weak primary health care system. The frail state of Nigerian primary health care places a heavy burden on tertiary hospitals. The problem is more pronounced in places where secondary care is also weak or mostly provided by the private sector. This results in inversion of the pyramidal distribution of patients such that the majority of patients are seen at the tertiary level. In response to this development, the GOPC in all Nigerian tertiary hospitals has family physicians who were trained to manage patients at primary and secondary care centres attending to these primary care patients. This makes the GOPC of a tertiary hospital in Nigeria a first contact facility for any type of patient.

The tertiary hospital where the study was conducted also serves as a referral centre for other lower cadre hospitals. The GOPC of the tertiary hospital is one of the primary care clinics of the hospital. It is run by consultant family physicians and resident doctors in Family Medicine. The general outpatient clinic (FOPC) of the hospital has a relatively large adult patient population and the health care providers take high numbers of BP measurements daily as part of the routine care of patients.

### Study design

This was a hospital-based, cross-sectional study.

### Study population

The study population comprised adult patients aged 18 years and above who attended the GOPC during the study period. A monthly average of 1168 patients was diagnosed at this clinic.

### Inclusion criteria

All adult patients aged 18 years and above with or without a prior history of hypertension were included in the study.

### Exclusion criteria

- Patients who had eaten within 30 min of BP assessment.
- Patients who had their BP checked for more than 10 min by the regular clinic nurses at the time of entering the consulting room.
- Patients who had smoked or taken coffee within 30 min of BP assessment.
- Patients with major psychiatric illness or severe illness who required urgent attention.

### Sample size

The formula for calculating sample size for a paired data was used,^13^ which is:

$$n=\frac{\delta_{{\text{d}}^{2}}{\left(\text{Z}\beta +\text{Z}\alpha /2\right)}^{2}}{{\text{difference}}^{2}}$$ [Eqn 1]

where:

*n* = sample size.

*d*_*d*_ = standard deviation (SD) of the within-pair difference. At 95% confidence interval (CI), a SD of ±0.34 was obtained as the SD of the differences between measurements obtained using the two methods (usual-care and guideline-concordant) from previous studies.^8,14^

difference = clinically meaningful difference between usual--care and guideline-concordant methods. A difference of 10 mmHg between the measurements from the two methods will be assumed to be a clinically meaningful difference. The mean usual-care systolic BP (SBP) and diastolic BP (DBP) values obtained from a previous study were 143 mmHg and 90 mmHg, respectively.^8^ A difference of 10 mmHg to the guideline-concordant BP will result in approximately 7% and 11% change in SBP and DBP, respectively. A 7% change was used as a clinically meaningful difference between usual-care and guideline-concordant methods because this will give a higher sample size.

Zβ = standard normal deviate that corresponds to power (80% power = 0.84)

Zα/2 = standard normal deviate that corresponds to a two-tailed significance level (1.96 for α = 0.05).

Therefore, $n=\frac{\delta_{{\text{d}}^{2}}{\left(\text{Z}\beta +\text{Z}\alpha /2\right)}^{2}}{{\text{difference}}^{2}}$

$$\begin{array}{l} n=\frac{0.34^{2}{\left(1.96+0.84\right)}^{2}}{0.07^{2}}\\ n=\frac{0.1156\times 7.84}{0.0049}\\ N=184.96. \end{array}$$

However, in order to allow for missing data, an attrition value (10% of the estimated minimum sample size) was added. The adjusted sample size (*n*1) is: *n*1 = *n*/(1−d) = 184.96/(1–0.1) = 184.96/0.9 = 205.5. This was approximated to 206.

### Sampling technique

A systematic random sampling technique was used to select 206 subjects attending the GOPC over a period of 1 month. With a monthly average of 1168 patients, the sampling interval was (1168/206) = 5.67. Therefore, every fifth patient presenting at the GOPC and who met the selection criteria was enrolled in the study. The first subject was selected by balloting once at the outset of the study after which every fifth eligible patient was recruited. The process was repeated on subsequent days until the sample size was achieved.

### Data collection and procedure

The exclusion criteria were ruled out through review of participants’ case notes and interviews. Information was obtained by the authors using an interviewer-administered questionnaire (see Appendix 2). The questionnaire had two sections: socio-demographic variables and clinical factors. The usual practice at our clinics is for the clinic nurses to conduct the BP measurement at the nursing station before patients see the doctor in the consulting room. In order to reduce bias that could arise from the clinic nurses changing their routine way of measuring BP because they are aware of the investigation underway and observer diagnostic suspicion bias (Hawthorne effect), they were blinded to the ongoing study.

Two research assistants who were registered nurses were specifically trained in guideline-concordant BP measurement methods using a protocol that followed JNC 7 recommendations using the same validated mercury sphygmomanometers that were available in the clinics for BP measurements.^15^ The sphygmomanometers were not labelled so that the sphygmomanometers could be used for guideline-concordant BP on one assessment day and for usual-care BP on another day to minimise the influence of equipment bias.

The usual-care BP was measured using the mercury sphygmomanometer employing the auscultation method by the regular four clinic nurses at the time of the study. Patients who were eligible for the study were recruited based on the sampling technique at this point. They were called into the consulting rooms to see the research-trained nurse assistants. Informed consent was obtained from eligible patients by the research nurse assistants. Participants had their BP re-assessed according to the standard protocol (The Seventh Report of the Joint National Committee on Prevention, Detection, Evaluation, and Treatment of High Blood Pressure [JNC 7] guideline) using the mercury sphygmomanometer employing the auscultatory method.^15^

The participant’s arm was bared up to the shoulder. Arm length was measured from the acromion to the olecranon process using a tape measure. The midpoint of the arm was marked using an eyebrow pencil. The arm circumference was measured horizontally at the midpoint mark by using the tape measure with the arm in a relaxed posture (to ensure the tape measure is at the proper tension). The appropriate size BP cuff was used based on the arm circumference (24 cm – 35.5 cm = medium cuff; 36 cm – 42 cm = large cuff; > 42 cm = extra-large cuff).^15,16^

The participant then sat comfortably in a chair with back support and both feet flat on the floor. The participant’s brachial artery was marked using an eyebrow pencil. The cuff was placed snugly on the arm with the inflatable inner bladder centred over the brachial artery and the lower edge of the cuff about 2.5 cm above the natural crease of the elbow. After the cuff had been properly placed, the participant was instructed to sit quietly without talking, eating, completing paperwork or crossing his or her legs for 5 minutes.

The cuff was inflated to 20 mmHg above that pressure at which the radial artery became impalpable. Systolic BP and DBP were measured via auscultation at the Korotkoff sound I and V. After a 2-min rest time, BP measurement was repeated. Between measurements, patients were asked to raise their arm for 5 seconds and rest their arm at the heart level for an additional 25 s to eliminate auscultation gap. The average of the two readings constituted the guideline-concordant BP.^15,16^ The procedure was repeated in the other arm. The arm with the higher average BP was used for guideline-concordant BP.

The time between usual-care and guideline-concordant BP assessment was kept to 10 min at the most so as to reduce the effect of time on BP values. Previous studies indicated that a time lag of fewer than 10 min does not have any significant effect on the BP value.^17^ The BP measurements were conducted between 10:00 and 15:00 on a daily basis. Finally, baseline demographic and clinical factors were obtained through interviews using a questionnaire (Appendix 2).

### Duration of the study

The study lasted for a period of 1 month.

### Statistical analysis

The data were analysed using the Statistical Package for Social Sciences (SPSS) version 21.0 program. Both descriptive and inferential statistics were used. For descriptive data, means ± SD values were used for continuous variable and percentages for categorical variables.

In a previously normotensive respondent or uncontrolled BP in a known patient with hypertension, BP ≥ 140/90 mmHg was considered as ‘hypertension’ in patients without diabetes or chronic kidney disease or BP ≥ 130/80 mmHg was considered as ‘hypertension’ in patients with chronic kidney disease or diabetes.^15^ The number of respondents with hypertension or uncontrolled BP in both guideline-concordant and usual-care was determined. Treatment decisions based on usual-care and standard BP were classified into: requires no treatment and requires treatment or needs adjustment of treatment. The proportion of respondents with the same treatment decision in usual-care and guideline-concordant treatment was calculated. In addition, the proportion of cases that would have been misclassified based on treatment decision using usual-care BP was determined.

The difference between means of SBP and DBP in the guideline-concordant and usual-care group was assessed using the paired *T*-test. Linear regression and Pearson’s correlation coefficient were used to determine the linear relationship between SBP and DBP values in the guideline-concordant and usual-care group. The Bland-Altman technique that is used for assessing agreement between two methods of clinical measurement was used to assess agreement between the two protocols of measurement.^14^ A difference of more than 10 mmHg between the measurements from the two methods was set as the clinically meaningful difference. The level of significance was set at a *p*-value of less than or equal to 0.05 and CI of 95%.

### Ethical considerations

The protocol was approved by the Health Research Ethics Committee of the hospital with protocol number FMCA/470/HREC/11/2016 and registration number FWA/Q0018660/02/28/2017. A sample of written consent is attached (see Appendix 1). The blinding of the clinic nurses in the usual-care protocol was done to eliminate the Hawthorne effect and ensure unbiased ascertainment of outcomes and maximise the validity of the results.

### Funding

The financing and sponsoring of the project were wholly at the expense of the researchers.

## Results

Two hundred and six participants were recruited for the study. The mean age of the respondents was 48.16 ± 14.45 years and 59.7% of them were female. The more frequently used arm for usual-care BP measurement was the left arm (59.7%). The range of the difference between guideline-concordant BP and usual-care BP was −24 mmHg to 90 mmHg for SBP and −38 mmHg to 44 mmHg for DBP (Table 1).

**TABLE 1:** Baseline demographic and clinical characteristics.

Variable	Category or range	Frequency (%) or mean ± SD
Age (years)	20–83	48.16 ± 14.45
Gender	Male	83 (40.3)
	Female	123 (59.7)
Arm used for usual-care BP	Right	89 (43.2)
	Left	117 (59.7)
Time between usual-care and guideline-concordant BP check (minutes)	2–10	7.84 ± 2.01
Mean usual-care systolic BP (mmHg)	70–220	123.22 ± 25.06
Mean guideline-concordant systolic BP (mmHg)	90–250	130.89 ± 24.97
Mean usual-care diastolic BP (mmHg)	40–150	74.62 ± 14.22
Mean guideline-concordant diastolic BP (mmHg)	40–136	81.76 ± 14.92
Mean of difference between guideline-concordant systolic BP and usual-care systolic BP (mmHg)	−24 to 90	7.67 ± 15.06
Mean of difference between guideline-concordant diastolic BP and usual-care diastolic BP (mmHg)	−38 to 44	7.14 ± 13.08

SD, standard deviation; BP, blood pressure.

**FIGURE 1:**
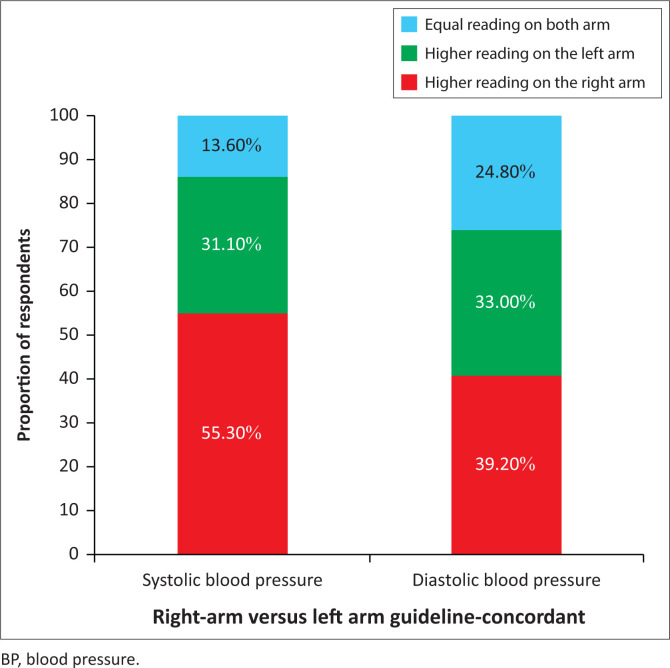
Guideline-concordant systolic and diastolic blood pressure measurement of both arms.

The guideline-concordant SBP and DBP readings were higher for the right arm than the left arm in most of the respondents (Figure 1).

**FIGURE 2:**
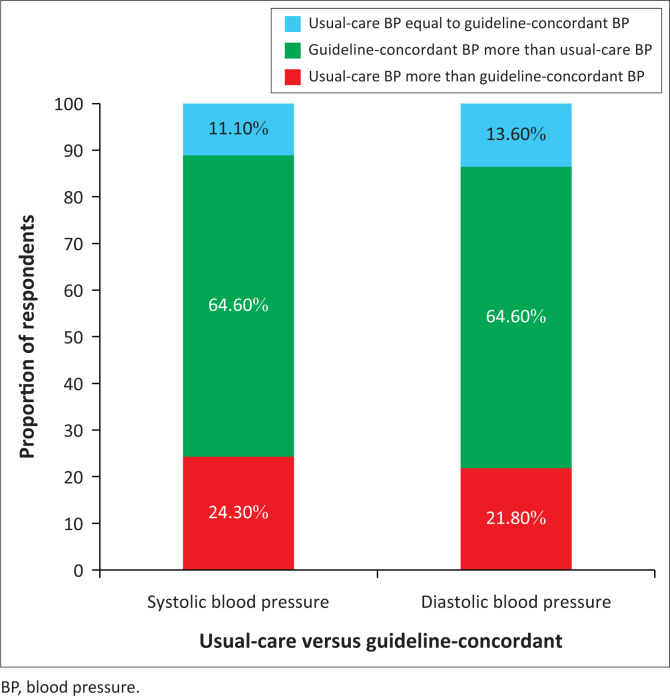
Comparison of usual-care and guideline-concordant blood pressure measurement.

Both guideline-concordant SBP and DBP measurements were greater than usual-care SBP and DBP measurements in 64.6% of the respondents (Figure 2).

The mean guideline-concordant SBP was 7.67 mmHg higher than the mean usual-care SBP (p ≤ 0.001), while the mean guideline-concordant DBP was 7.14 mmHg higher than the mean usual-care DBP (p ≤ 0.001) (Table 2).

**TABLE 2:** Comparison of means of blood pressure (systolic and diastolic) between guideline-concordant and usual-care blood pressure measurement protocols using the paired *T*-test.

Variable	Category	Mean ± SD	*T*	Sig.
Systolic BP	Guideline-concordant	130.89 ± 24.97	7.31	< 0.001
	Usual-care	123.22 ± 25.06	-	-
Diastolic BP	Guideline-concordant	81.76 ± 14.92	7.83	< 0.001
	Usual-care	74.62 ± 14.22	-	-

SD, standard deviation; BP, blood pressure. Sig., significance.

The proportion of participants classified as having hypertension among respondents who were not previously hypertensive was 11.8% lower when using usual-care BP compared to guideline-concordant BP (16.8% vs. 28.6%). The proportion of participants classified as having uncontrolled BP among respondents who previously had hypertension was 15% lower when using usual-care BP compared to guideline-concordant BP (44.8% vs. 59.8%) (Table 3).

**TABLE 3:** Pattern of blood pressure among respondents based on usual-care and standard blood pressure measurement protocols.

Blood pressure control based on usual-care BP	Blood pressure control based on guideline-concordant BP									
	Respondents who previously had no hypertension					Respondents who previously had hypertension				
	Normal BP		Hypertension		Total	Controlled BP		Uncontrolled BP		Total
	*N*	%	*N*	%		*N*	%	*N*	%	
	82	82.8	17	17.2	99	27	56.3	21	43.8	48
	3	15.0	17	85.0	20	8	20.5	31	79.5	39
**Total**	**85**	**71.4**	**34**	**28.6**	**119**	**35**	**40.2**	**52**	**59.8**	**87**

BP, blood pressure.

Overall, the treatment decisions based on the two protocols were in agreement in 155 (75.2%) of the respondents; hence, 51 (24.8%) respondents had incorrect treatment diagnosis based on usual-care BP measurement. Of the participants who were not supposed to start or have treatment adjustment based on the guideline-concordant measurement protocol, 13 (22.0%) of them would have been wrongly given antihypertensives based on the usual-care BP measurement protocol. Of those who will need to start or change treatment based on the guideline-concordant measurement protocol, 38 (25.9%) of them would have missed the opportunity if the treatment decision was based on the usual-care BP measurement protocol (Table 4).

**TABLE 4:** Comparison of treatment decisions based on usual-care and guideline-concordant blood pressure measurement protocols.

Treatment plan based on usual-care blood pressure measurement protocol	Treatment plan based on usual-care BP measurement protocol				Total
	No treatment		Treat or change treatment		
	*N*	%	*N*	%
No treatment	109	74.1	38	25.9	147
Treat or change treatment	13	22.0	46	78.0	59
**Total**	**122**	**59.2**	**84**	**40.8**	**206**

BP, blood pressure.

The intra-class coefficient (ICC) of 0.87 (95% CI: 0.77–0.93) was almost perfect for SBP and strong for DBP; ICC = 0.70 (95% CI: 0.48–0.81) for the two BP protocols. The linear regression relationship for SBP and DBP between usual-care (pragmatic) and guideline-concordant (standardised) BP was summarised as SBPPr = 15.68 + 0.82 × SBPSt and DBPPr = 28.06 + 0.57 × DBPSt (Table 5).

**TABLE 5:** Pearson (*r*), intra-class coefficient and regression equations for blood pressure measurement methods.

Blood pressure	BP measurement method in comparison	Pearson coefficient, *r* († interpretation)	Intra-class coefficient (‡ interpretation)	Regression equations for relationship between blood pressure methods
Systolic BP	Guideline-concordant/usual-care	0.8	0.9	SBPPr = 15.68 + 0.82 × SBPSt
Diastolic BP	Guideline-concordant/usual-care	0.6	0.8	DBPPr = 28.06 + 0.57 × DBPSt

BP, blood pressure; SBPPr, systolic blood pressure pragmatic; SBPSt, systolic blood pressure standardised; DBPPr, diastolic blood pressure pragmatic; DBPSt, diastolic blood pressure standardised.

† , Interpretation based on Pearson coefficient (r): −1.0 to −0.7 strong negative association; −0.7 to −0.3 weak negative association; −0.3 to +0.3 little or no association; +0.3 to +0.7 weak positive association; +0.7 to +1.0 strong positive association.

‡ , Interpretation based on intra-class coefficient: Intra-class coefficient can be interpreted as follows: 0.0–0.2 indicates poor agreement: 0.3–0.4 indicates fair agreement; 0.5–0.6 indicates moderate agreement; 0.7–0.8 indicates strong agreement, and > 0.8 indicates almost perfect agreement.

**FIGURE 3:**
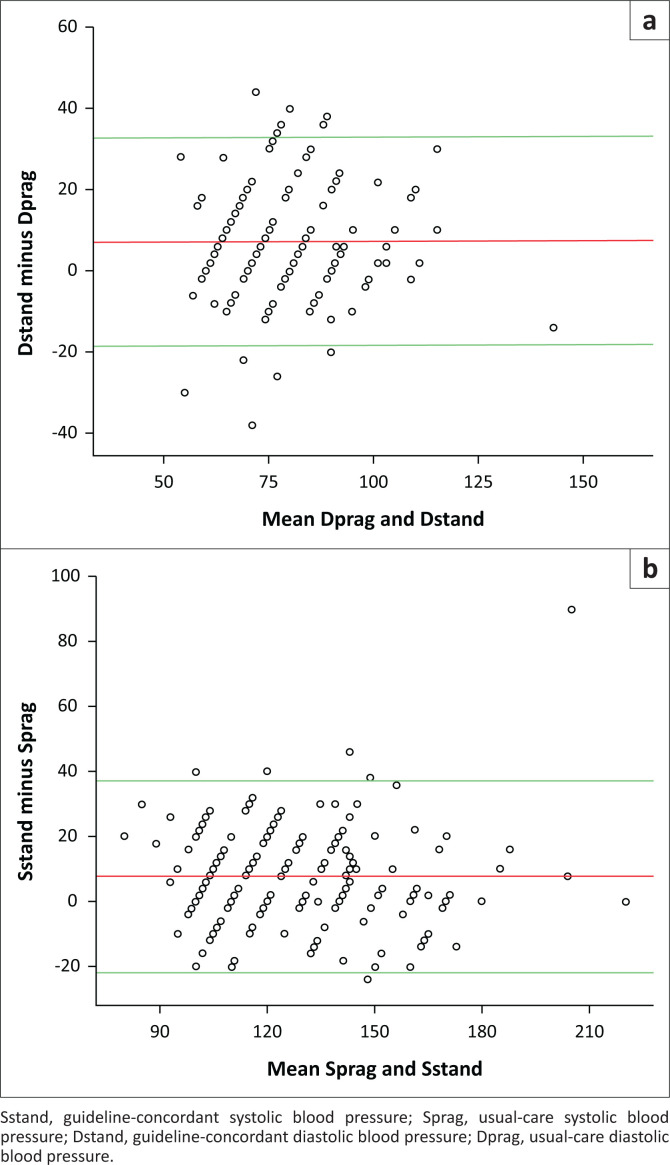
The Bland-Altman plot difference against the mean for (a) diastolic blood pressure and (b) systolic blood pressure – guideline-concordant compared to usual-care.

From the Bland-Altman plot, the limits of agreement between the usual-care and standardised BP were between −21.85 mmHg and 37.19 mmHg for SBP and −18.51 mmHg and 32.78 mmHg for DBP. The two methods could not be used interchangeably because the pre-defined maximum allowed difference of 10 mmHg was smaller than the higher limits of agreement and higher than the lower limits of agreement (Figure 3a and b).

## Discussion

The study showed that the left arm was used for the single BP measurement in about two-thirds of the participants in the usual-care protocol. Anecdotal evidence from the regular clinic nurses that measured the usual-care BP showed that the BP measurement on the left arm is more reliable because of its closeness to the heart. This may not always be true as seen in the index study where the SBP and DBP readings on the right arm were higher than those of the left arm in the majority of the participants. The preferred choice of the left arm for BP measurement by the nurse in the usual-care protocol contradicts the recommendation of various guidelines on BP measurement which state that the arm with higher reading should be used for measuring the BP.^4,15,16^ Thus, the belief that left arm BP will be more reliable because of its proximity to the heart may lead to misdiagnosis of hypertension because there is a chance of higher BP reading in either arm. This underscores the importance of guidelines’ recommendation of dual-arm BP measurements at the initial visit, and subsequent measurement of BP in the arm with higher reading.^4,15,16^

Our study showed that the mean SBP and DBP values measured using the usual-care measurement protocol were lower compared with the those using the guideline-concordant measurement protocol. The reason for the lower usual-care BP values in the index study may be attributed to non-adherence to BP measurement guidelines. For instance, the usual-care BP measurement protocol that was solely based on the readings from one arm, in most cases the left arm in the index study, may lead to lower usual-care BP values because of the evidence in favour of higher BP on the right arm when using the guideline-concordant protocol in the majority of the participants.

Similar to this study, a previous Canadian study that compared casual in-clinic BP measurements to standardised BP measurements among severely obese patients in a bariatric clinic also found that casual in-clinic BP measurements were, on average, lower than standardised measurements.^18^

Unlike the index study and the Canadian study, the previous studies on this topic, however, reported contrary conclusions; that is, usual-care BP measurements were higher than standardised measurements.^7,8,19,20^ A South African study showed that the mean usual-care SBP and DBP were significantly higher than mean guideline-concordant SBP and DBP by 10.7 mmHg and 3 mmHg, respectively.^8^ Similarly, Sewell et al. also showed that usual-care BP values frequently tended to be higher than the guideline-concordant BP values.^7^

Ideally, higher readings in the usual-care BP will be expected because most of the reasons for inaccurate BP measurement that are common with usual clinic BP measurement result in falsely elevated readings. The potential bias would be more in the Canadian study where severely obese patients were used because of the recognised concerns related to arm circumference, length and shape which can predispose to falsely elevated readings.

The potential explanation for the observed difference in findings between the index study or Canadian study and previous studies^7,8,19,20^ concerning the variation between usual-care and guideline-concordant BP can be ascribed to the different BP measurement protocol that was used in the guideline-concordant measurement protocol. While our study and the Canadian study^18^ employed dual-arm BP measurement and used the arm with higher reading for the guideline-concordant BP, the previous studies^7,8,19,20^ used the readings from a single arm as the guideline-concordant value. The use of a single arm for measuring guideline-concordant BP in the previous studies^7,8,19,20^ is not in conformity with BP measurement guidelines that stipulated dual-arm BP measurements at the initial visit and subsequent measurement of BP in the arm with higher reading.^4,15,16^ This may result in lower guideline-concordant BP measurement if the arm used was the one with lower BP readings. The selective use of one arm for guideline-concordant BP protocol could introduce bias that may affect the validity. This suggests that researchers working on BP-related protocol must follow all the BP measurement recommended guidelines to aid comparability and avert misleading results on various indices of cardiovascular health. There is a need for further studies on this topic with emphasis on dual-arm BP measurement when using the standardised BP protocol.

The implications of the significant variation between usual-care and guideline-concordant BP on diagnosis was obvious in the index study. Our study showed that the usual-care BP measurements underestimated BP, reducing the diagnosis of uncontrolled hypertension by 15% in previously hypertensive patients and hypertension by 11.8% in previously normotensive patients. This observation was similar to many studies that showed misdiagnosis of hypertension if BP was not measured according to guidelines.^7,8,19^ This measurement bias may deprive patients of effective antihypertensive therapy in preventing cardiovascular morbidity and mortality.

The misdiagnosis based on usual-care BP measurement in this study affected the treatment decision. About one out of every four (24.8%) participants in the study had an incorrect treatment diagnosis. Majority of the participants who had incorrect treatment decision would have missed the opportunity of being treated or get exposed to unnecessary adverse effects of these drugs based on usual-care BP. It is obvious that the improvement in BP measurement techniques might result in the prevention of needless mortality and treatment burden.

This study showed that the use of the correlation coefficient to see whether usual-care protocol agrees with the guideline-concordant protocol for BP measurement was misleading. Despite the strong correlation coefficient, the Bland-Altman plot showed that the two protocols did not agree. This lack of agreement is by no means obvious from our findings with the limits of agreement being wider than the within 10 mmHg clinical reference range. The difference is enough to even affect decisions on patients’ management even if the reference ranges for hypertension severity grading using JNC 7 guideline-based value of 20 mmHg for SBP and 10 mmHg for DBP were used.^15^ This implies that the two protocols of BP measurement could not be used interchangeably and were not measuring the same thing. This finding further reinforces the importance of thorough adherence to the BP measurement protocol.

The use of ambulatory and automated measurement of BP in clinical practice and research is likely to eliminate this disparity and its consequences because of reliability and consistency of diagnosis of hypertension.^21^ The high cost and non-availability of ambulatory monitoring and automated measurement of BP in Nigeria makes careful training of primary health workers a feasible option for now. The strict adherence to BP measurement protocols by trained staff has been shown to result in manual BP measurements that correlate much better with ambulatory readings.^21^ Therefore, the relevance of adequate training and retraining of health workers on BP measurement guidelines cannot be understated.

## Conclusion

This study showed that the usual-care BP readings varied significantly from the guideline-concordant BP readings. Furthermore, we unexpectedly found that the usual-care BP protocol might underestimate the BP readings obtained from the guideline-concordant protocol. The incongruence between usual-care BP readings and guideline-concordant BP readings could result in an incorrect diagnosis and inappropriate treatment. The training of primary health care workers who are involved in BP measurements will improve the level of adherence to BP measurement guidelines and eliminate incorrect BP measurement and its consequences.

### Limitations

The regular clinic nurses who measured the usual-care BP may not accurately represent how other health workers will adhere to BP measurement guidelines. In addition, the BP measurements may be elevated in the presence of health care professionals – the white coat effect. However, the measurement of BP by nurses using both usual-care and guideline-concordant protocols makes it negligible because the white coat effect appears to be greater for doctors than for nurses.^22^ Furthermore, we cannot totally affirm that the blinding of the clinic nurses in the usual-care measurement was absolute as there was a possibility of discussion with the research-trained nurses on the guideline-concordant arm outside the study site. The circadian rhythm of BP characterised by an early morning surge until it reaches peak around noon time can lead to overestimation of the proportion of patients labelled to be hypertensive in this study. Lastly, the on-the-spot diagnosis of hypertension in the study might have over-diagnosed hypertension as the 6-h interval was not used.

## Appendix 1: Informed Consent

Dear Sir/Ma,

I hereby seek your consent to participate in this research.

I am a doctor at the Department of Family Medicine, Federal Medical Centre, Abeokuta. I intend to find out the variation between usual-care blood pressure measurement and guideline-concordant blood pressure measurement among patients seen at a family practice clinic of the Federal Medical Centre, Abeokuta- Implications for subsequent management.

If you consent, a questionnaire will be administered on you followed by a physical examination. The procedure will last for about 30 minutes. The result of this project will be published in a journal.

Your participation is entirely of your own free will and you can withdraw from the study at any time you like without explanation. Refusal to participate in the study will not affect your treatment in anyway. You have the right to refuse to answer any question you don’t want to answer.

Please note that any information collected will remain confidential. Your name will not be attached to any published results. Kindly indicate your decision by signing in the space below.

Thank you.

Date and signature or thumb print of witness    Date and signature or thumbprint of participant

## Appendix 2: Questionnaire

**RESEARCH PROFORMA ON ANALYSIS OF VARIATION BETWEEN USUAL-CARE AND GUIDELINE-CONCORDANT BLOOD PRESSURE (BP) MEASUREMENTS AMONG PATIENTS SEEN AT A FAMILY PRACTICE CL INIC IN WESTERN NIGERIA – IMPLICATIONS FOR SUBSEQUENT MANAGEMENT.**

Good day Sir/Ma,

Thank you for consenting to participate in this study. This research is about finding out the variation between usual-care and guideline-concordant blood pressure measurements. It will help us serve you better. Your cooperation is needed to truthfully answer the questions below. All information will be strictly confidential and it will take only a few minutes. Thank you.

Serial Number …………………………………..

Hospital number ………………………………..

Date …………………………………………………

- **SOCIO- DEMOGRAPHIC VARIABLES**
  1. **Age** ………..… years
  2. **Gender:**
    - Male
    - Female
  3. **Marital status:**
    - Single
    - Married
    - Divorced
    - Separated
    - Widowed
  4. **Religion:**
    - Islam
    - Christianity
    - Traditional belief
    - Others
  5. **Ethnic group:**
    - Yoruba
    - Hausa
    - Igbo
    - Others
  6. **Level of education completed by subject:**
    - No formal education
    - Primary
    - Secondary
    - Tertiary
- **CLINICAL FACTORS**
  7) **Time for usual-care blood pressure   …………**
  8) **Time for guideline-concordant blood pressure ……………**
  9) **Which arm did they use for checking your blood pressure (usual-care)?**
    - Right arm
    - Left arm
    - Both arm
  10) **Blood pressure based on usual-care and guideline-concordant BP**
    **Table:**
    BP		Usual-care BP readings	Guideline-concordant BP readings	Difference Guideline-concordant and usual-care BP readings
    SBP	Right arm			
    	Left arm			
    DBP	Right arm			
    	Left arm			
  11) **Tick as appropriate based on the blood pressure value above**
    **Table:**
    	Guideline-concordant	Usual-care
    Normal		
    Controlled		
    Uncontrolled		
    Hypertension		
  12) **Treatment decision class based on usual-care and guideline-concordant BP**
    **Table:**
    Treatment decision	No treatment	Treat	Change treatment
    Based on usual-care BP			
    Based on guideline-concordant BP			
  13) **Would this patient treatment outcome have been misclassified based on the usual-care BP only?**
    - Yes
    - No
  14) **Treatment outcome comparing usual-care BP and guideline-concordant BP treatment decision**
    **Table:**
    Classification	Yes	No
    Wrongly treated with anti-hypertensives based on usual-care BP measurement only		
    Missed the opportunities of being treated based on usual-care BP		
